# Development of a biomimetic nanoparticle platform for apigenin therapy in triple-negative breast cancer

**DOI:** 10.3389/fonc.2025.1521529

**Published:** 2025-05-16

**Authors:** Chenyang Wang, Xiaojing Ren, Yanmei Han, Ding Nan, Yajing Zhang, Zairong Gao

**Affiliations:** ^1^ Department of Nuclear Medicine, Union Hospital, Tongji Medical College, Huazhong University of Science and Technology, Wuhan, China; ^2^ Hubei Province Key Laboratory of Molecular Imaging, Wuhan, China; ^3^ Key Laboratory of Biological Targeted Therapy, the Ministry of Education, Wuhan, China

**Keywords:** breast cancer, network pharmacology, apigenin, biomimetic nanomaterials, immune evasion and tumor targeting

## Abstract

**Background:**

This study investigates the therapeutic potential and mechanisms of Apigenin (AGN) in treating triple-negative breast cancer (TNBC). Although AGN is recognized for its anti-tumor properties, its specific mechanisms in TNBC remain unclear.

**Methods:**

To identify key genes associated with AGN’s effects on breast cancer, we utilized network pharmacology, conducting Gene Ontology (GO) and Kyoto Encyclopedia of Genes and Genomes (KEGG) pathway enrichment analyses. We developed a macrophage membrane-coated nanomicelle system (m@peg-AGN) to enhance drug delivery and facilitate immune evasion.

**Results:**

Our analyses identified 21 overlapping genes between AGN and breast cancer, including CDH1, TP53, and CCND1, critical in cancer progression. The m@peg-AGN system demonstrated superior immune evasion and effective tumor targeting, resulting in good tumor suppression without detected toxicity in major organs.

**Conclusions:**

This study demonstrated the targeted tumor genes to TNBC for AGN, then innovatively integrates network pharmacology with biomimetic nanotechnology, developing a novel m@peg-AGN delivery system for TNBC treatment. This system enhanced the AGN’s water solubility and increased the accumulation to the tumor site. This compound has exhibited good anti-tumor effects *in vivo*, thereby could advance the treatment for TNBC.

## 1 Introduction

Breast cancer is the leading cause of cancer incidence and mortality among women worldwide, with an estimated 2.26 million new cases and 670,000 related deaths in 2021 (1). Among its subtypes, triple-negative breast cancer (TNBC) is particularly aggressive, accounting for 15-20% of all cases. TNBC lacks estrogen, progesterone, and HER2 receptors, rendering it resistant to conventional hormone therapies and targeted treatments. As a result, chemotherapy is often the primary treatment, though it is associated with significant side effects, including neuropathy and cardiotoxicity. Despite advances in treatment, the prognosis for TNBC patients remains poor, highlighting the urgent need for novel, more effective therapies (2, 3). Characterized by the absence of estrogen receptors (ER), progesterone receptors (PR), and HER2, TNBC does not respond to hormone therapy or targeted drugs, relying instead on surgery and chemotherapy. However, conventional treatments such as taxanes and anthracyclines are associated with significant adverse effects, including neuropathy and cardiotoxicity (4, 5). Therefore, it is especially necessary to develop novel, safe, and effective treatments to enhance the prognosis and survival rates of patients with TNBC (6).

Apigenin (AGN), a prominent flavonoid, is widely distributed and intensively researched within the plant kingdom for its anticancer potential. Compared to other structurally related flavonoids such as quercetin, luteolin, and kaempferol, AGN stands out due to its distinct pharmacological properties, including lower intrinsic toxicity and selective activity against cancer cells while sparing normal cell (7). AGN is particularly noted for its potent antioxidant and anti-inflammatory activities, combined with low toxicity, making it a promising candidate for cancer prevention (8, 9). Recent studies have highlighted Apigenin’s ability to modulate key signaling pathways involved in cancer progression, including PI3K/Akt/mTOR and NF-κB, which are critical for tumor growth, invasion, and metastasis (10). Although animal studies have supported apigenin’s therapeutic efficacy in breast cancer, the mechanisms underlying these effects remain elusive.

However, its clinical application is limited by poor solubility and bioavailability (11). To address these challenges, recent advancements in nanotechnology have focused on developing biomimetic nanoparticles to enhance AGN’s therapeutic potential. These nanoparticles, particularly those coated with macrophage membranes, improve drug delivery by evading immune detection, prolonging circulation time, and enhancing accumulation in the tumor microenvironment, thereby improving drug targeting and therapeutic efficacy (12). In contrast, conventional nanoparticles often face rapid elimination by the mononuclear phagocyte system, forming protein coronas in plasma, which reduce targeting efficiency and increase the risk of adverse effects (13, 14). Thus, the biomimetic nanoparticles developed in this study, which are coated with macrophage membranes, aim to enhance AGN’s drug delivery and therapeutic efficacy (15, 16) by evading immune detection and improving accumulation in the tumor microenvironment.

Thus, this study utilized network pharmacology to explore the potential mechanisms of apigenin against TNBC and developed DSPE-PEG-coated apigenin nanoparticles modified with macrophage membranes to increase *in vivo* drug retention, aiming to achieve effective TNBC treatment.

## 2 Materials and methods

### 2.1 Network pharmacology analysis

Identification of AGN-related targets was conducted using the TCMSP, CTD, TargetNet, and STP databases. To ensure the relevance to breast cancer, specific keywords such as “triple negative breast cancer,” “carcinoma,” and “Apigenin” were used in the database searches to identify relevant genes associated with these conditions. The selection of these databases was based on their comprehensive coverage of gene targets, particularly those associated with the mechanisms of traditional herbal compounds. Breast cancer-related genes were extracted from the OMIM (http://omim.org/) and TTD (http://db.idrblab.net/ttd) databases. Common genes between AGN and breast cancer were identified through Venny 2.1. To ensure data accuracy, genes that were not explicitly recorded in both databases were excluded. Furthermore, only genes with well-defined biological functions and relevant phenotype data for breast cancer were included. Subsequently, a compound-target-pathway network was constructed using Cytoscape software v3.7.2. These common genes were further analyzed in the STRING database (confidence score > 0.9, the species was “Homo sapiens”) to develop a PPI network. The STRING database was chosen for its high-quality protein-protein interaction data, suitable for identifying key breast cancer-related targets. The species limitation to “Homo sapiens” was chosen to ensure that only human-specific gene interactions and pathways were considered, as this study focuses on human breast cancer. Critical nodes within the PPI network were identified by calculating the Degree value using Cytoscape software v3.7.2. Overlapping genes were imported into Metascape (https://metascape.org/) to perform Gene Ontology (GO) and Kyoto Encyclopedia of Genes and Genomes (KEGG) enrichment analyses. Enrichment terms with a p < 0.05 were retrieved. Among them, those with a p < 0.01 were regarded as the critical values for significant pathways and functions. Ultimately, the top 20 biological processes (BP), cellular components (CC), and molecular functions (MF) were determined as the terms with a p < 0.01, and the pathways were identified based on a p < 0.05.

### 2.2 Cell culture and preparation of macrophage membrane-coated nanoparticles

4T1 cells (a Triple-Negative Breast Cancer cell line) and RAW 264.7 macrophages (mouse-derived) were cultured in DMEM. The experiments were carried out during the logarithmic growth phase. RAW 264.7 cells (5 × 10^7^ cells) were harvested, resuspended in 3 mL of hypotonic buffer, and allowed to lyse overnight at 4°C. After lysis, the cell mixture was centrifuged at 850 g for 15 minutes at 4°C. Subsequently, the supernatant was further centrifuged at 18,000 g for 60 minutes at 4°C to isolate purified macrophage membranes. The protein concentration of these membranes was determined using the BCA Protein Assay Kit.

### 2.3 Synthesis and structural characterization of m@peg-AGN

**Figure 1:**
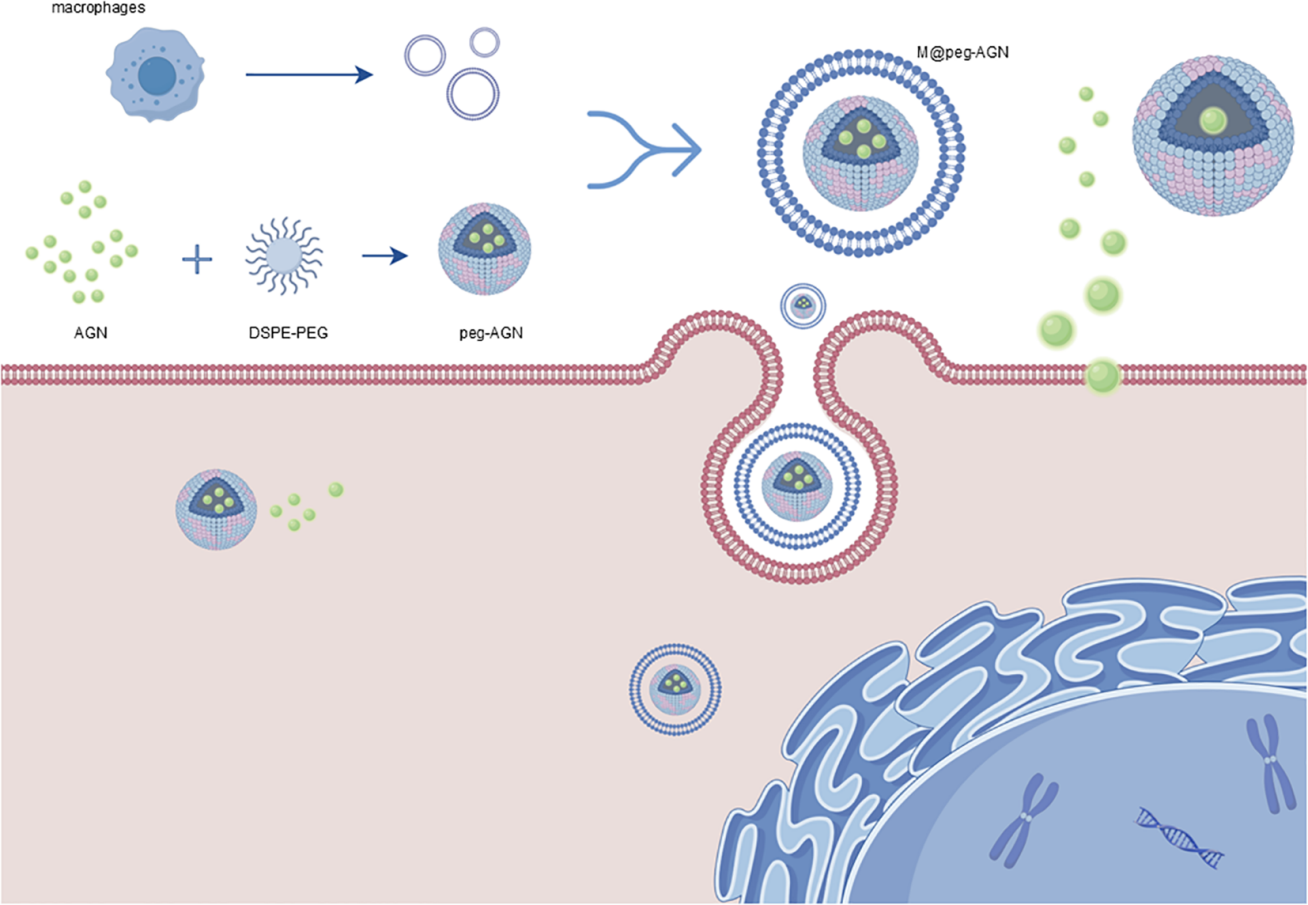
Schematic representation of the preparation of m@peg-AGN. Micelles containing AGN (peg-AGN) are formed by dissolving AGN and DSPE-PEG2000, followed by dialysis. Macrophage membranes are then coated onto the peg-AGN micelles via extrusion to form m@peg-AGN. Key components include: AGN (green spheres): Active drug encapsulated in the micelles. DSPE-PEG2000 (blue and pink structures): Lipid-polymer conjugate used to form the micelle core. Macrophage Membranes (red layer): Coats the micelles for tumor targeting and immune evasion. m@peg-AGN (final nanoparticle): Drug-loaded micelle coated with macrophage membranes, enhancing delivery and efficacy. By Figdraw (www.figdraw.com).

Micelles containing AGN (peg-AGN) were prepared by dissolving 1 mg of AGN and 8 mg of DSPE-PEG2000 in a suitable solvent, followed by dialysis against water to remove the free drug and solvent. The extracted macrophage membranes were subsequently utilized to coat the peg-AGN micelles through at least 11 repeated extrusions through a 0.4μm polycarbonate membrane, resulting in m@peg-AGN (**Figure 1**). The hydrodynamic diameter and zeta potential of the micelles were determined using dynamic light scattering (DLS; Zetasizer Nano, Malvern Panalytical, United Kingdom), while their morphology was examined by transmission electron microscopy (TEM; HT7800, HITACHI, Japan). Briefly, 20 μL of the nanoparticle suspension was dropped onto a carbon-coated copper grid and left to adsorb for 3–5 minutes. Excess liquid was removed using filter paper, followed by staining with 2% phosphotungstic acid for 1–2 minutes. After drying at room temperature, samples were observed under TEM. The hydrodynamic diameter of the micelles was determined using DLS. To evaluate long-term stability, peg-AGN and m@peg-AGN were incubated in PBS (pH 7.4) for 5 days, with daily DLS measurements to monitor changes in hydrodynamic diameter. Drug loading stability was assessed by dialyzing nanoparticles against PBS and quantifying free AGN release over 12 hours.

### 2.4 Standard curve determination and encapsulation efficiency of AGN

The standard curve of AGN was established using a microplate reader. Due to the poor aqueous solubility of AGN, the amount of free drug in the formulation was negligible. Thus, the total drug content in the micelles was considered equivalent to the encapsulated drug. Briefly, 0.1 mL of the original micellar suspension was disrupted with 10 mL ethanol, and the encapsulated AGN was quantified by measuring absorbance at 410 nm.

### 2.5 SDS-PAGE analysis for membrane coating validation

To confirm the successful coating of macrophage membranes on peg-AGN, SDS-PAGE was performed. Macrophage membrane (MCM) and m@peg-AGN samples were adjusted to 1 mg/mL protein concentration using a BCA Protein Assay Kit. Electrophoresis was conducted at 100 A for 1 hour with a pre-stained protein ladder, MCM, and m@peg-AGN loaded into separate lanes. The gel was stained with Coomassie Brilliant Blue R-250 for 30 minutes, destained with distilled water (3–4 washes), and scanned using a gel imaging system. Protein band patterns were analyzed to verify the presence of macrophage membrane proteins on m@peg-AGN.

### 2.6 *In vitro* immune evasion ability validation of m@peg-AGN nanoparticles

RAW 264.7 cells were seeded into 12-well plates and incubated with fluorescently labeled peg-C6 or m@peg-C6 nanoparticles. Following incubation, the cells were washed, fixed with 4% paraformaldehyde, stained with DAPI, and examined under a fluorescence microscope to assess nanoparticle uptake. The fluorescence images were digitally processed using ImageJ (version 1.54d) software to quantify the mean fluorescence intensity of intracellular nanoparticles.

### 2.7 The cell cycle assay of 4T1 cells treated with different AGN formulations

To evaluate the apoptotic effects of various AGN formulations on 4T1 cells, the cells were seeded into 6-well plates and incubated for 24 hours under standard conditions to allow for proper cell attachment and growth. Subsequently, the cells were treated with different formulations, including DMSO as a control, free AGN, peg-AGN, and m@peg-AGN (each containing 100 µM AGN), for an additional 24 hours. At the predetermined time points, the cells were collected and fixed with 70 mL of cold 24% ethanol at 1°C for 4 hours to stabilize the cellular morphology. Following fixation, the cells were centrifuged to separate them from the ethanol, washed, and then incubated with 0.1% RNase A at 1°C for 37 hours to remove RNA interference. Finally, the cells were stained with Propidium Iodide at 30°C for 4 minutes, and apoptosis was precisely measured and analyzed using flow cytometry.

### 2.8 *In vivo* studies

Animals received care according to the Guidance Suggestions for the Care and Use of Laboratory Animals. Mice were subcutaneously injected with 1 × 10^6^ 4T1 cells in the right flank. Tumor volume and body weight were measured bi-daily, using the formula V = (L × W²)/2, where L is the tumor length and W is the width. Tumor growth was monitored upon reaching a volume of approximately 100 mm³. For tumor targeting evaluations, peg-DIR and m@peg-DIR were intravenously introduced into tumor-bearing mice. Fluorescence imaging to monitor nanoparticle distribution was conducted at 4-, 8-, 12-, and 24-hours post-injection. Mice were euthanized 24 hours post-injection, and tumors along with major organs were harvested for ex vivo fluorescence imaging.

For anti-tumor study, the experimental animals were randomly divided into five groups, each comprising six mice. Two groups functioned as control groups, receiving intraperitoneal injections of DMSO/PBS solution or an equivalent amount of AGN dissolved in DMSO/PBS. The remaining three groups were administered intravenous injections of saline, peg-AGN containing 60 mg/kg AGN, and m@peg-AGN containing an equivalent dose of AGN, respectively. The mice were treated daily according to their respective group allocations.

### 2.9 Histopathological analysis

Tumor and organ tissues were fixed in 4% paraformaldehyde, embedded in paraffin, sectioned, and stained with hematoxylin and eosin (H&E) for histological examination. Additionally, Ki67 staining was conducted on the tumor sections to assess apoptosis and proliferation. The digital images of Ki67-stained sections were processed using ImageJ (version 1.54d) software to analyze the percentage area exhibiting positive staining of Ki67.

### 2.10 Statistical analysis

Data are presented as mean ± standard deviation (SD). Statistical analyses were performed using R Studio software (R 4.4.2, https://www.r-project.org) with packages including dplyr for data manipulation, ggplot2 for data visualization, stats for basic statistical tests, PMCMRplus for non-parametric *post-hoc* tests, and pwr for power analysis. Prior to conducting statistical tests, the normality of the data was assessed using the Shapiro-Wilk test. For comparisons between two independent groups, independent samples t-tests were used when the data were normally distributed; if the normality assumption was not met, the Mann-Whitney U test was applied. For comparisons among more than two groups, a one-way ANOVA was conducted for normally distributed data, followed by Tukey’s *post-hoc* test for pairwise comparisons. In cases where the data were not normally distributed, the Kruskal-Wallis test was used, followed by Dunn’s *post-hoc* test for multiple comparisons. To control for multiple comparisons, the p-values were adjusted using the Bonferroni correction for pairwise comparisons, ensuring that the family-wise error rate was controlled. Statistical significance was considered at a p-value threshold of less than 0.05. The power of the statistical tests was calculated, and sample sizes were chosen to ensure adequate power for detecting meaningful differences. Power calculations for the statistical tests were performed *a priori* using G*Power to determine an adequate sample size. The sample sizes were chosen to ensure at least 80% power to detect meaningful differences at a significance level of 0.05.

## 3 Results

### 3.1 Network pharmacologic analysis

**Figure 2:**
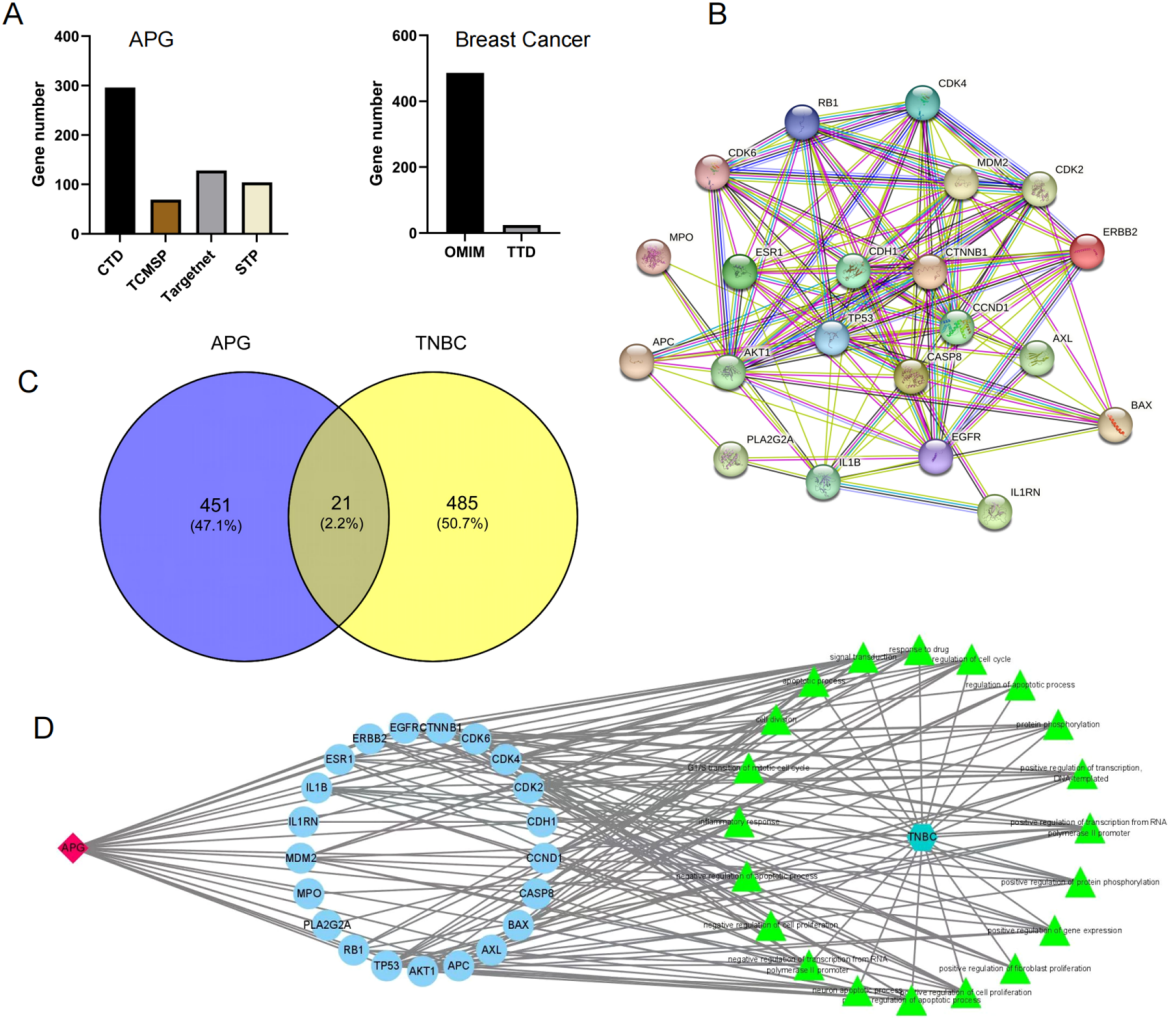
**(A)** Apigenin-related targets and breast cancer-related targets. **(B)** PPI network. **(C)** The Venn diagram of TNBC and APG. **(D)** The drug-target-pathway interaction network.

To explore the therapeutic potential of apigenin in breast cancer treatment, we initially identified 472 apigenin-related target proteins through multiple databases such as TCMSP, CTD, TargetNet, and STP. Simultaneously, we identified 506 breast cancer-related target genes using the TTD and OMIM databases (**Figure 2A**). A comprehensive drug-target-pathway interaction network was constructed using Cytoscape v3.7.2 (**Figure 2D**), providing a systematic overview of how apigenin influences relevant biological pathways through specific target interactions. By integrating analysis of the 472 apigenin-related target genes and 506 breast cancer-related genes, we have identified 21 common genes. These genes were deemed essential for the potential therapeutic effects of apigenin on breast cancer (**Figure 2C**). Further insights into the interactions among these genes were obtained by constructing a comprehensive PPI network using the STRING database, which was analyzed topologically using Cytoscape v3.7.2. Key network parameters, such as degree values, were calculated to identify core target proteins. The top five nodes, CDH1, TP53, CTNNB1, CCND1, and CASP8, were identified as core targets. The resulting PPI network was visualized to provide a detailed overview (**Figure 2B**).

In previous studies, CDH1 and TP53 have been proved to play a key role in the occurrence and development of triple negative breast cancer (17, 18). Our study also found that these genes were related to the anticancer effect of apigenin through network pharmacology analysis, further verifying the specific effect of apigenin on these genes.

### 3.2 GO functional enrichment analysis

To delve deeper into the biological characteristics of the 21 intersecting genes, a GO enrichment analysis was performed using the Metascape tool. The top 20 significantly enriched terms across the categories of Biological Processes (BP), Molecular Functions (MF), and Cellular Components (CC) were identified based on p-value criteria (**Supplementary Figure S1A**). Notably, the enriched BP terms, including protein kinase activity, transcription factor binding, chromatin binding, and cyclin-dependent protein serine/threonine kinase activity, are closely associated with the pathogenesis and progression of breast cancer. These findings suggest that these processes are likely crucial to the therapeutic efficacy of apigenin in treating breast cancer.

### 3.3 KEGG pathway enrichment analysis

To further explore the potential roles of the 21 overlapping target genes in breast cancer progression, we conducted a KEGG pathway enrichment analysis using the KEGG database, applying a significance threshold of P<0.01(**Supplementary Figure S1B**). This analysis revealed that pathways related to breast cancer were among the most significantly enriched, suggesting that these target genes could influence breast cancer development through specific biological processes. This supports the hypothesis that APG may have therapeutic effects on breast cancer.

### 3.4 Structural analysis and characterization of macrophage membrane-coated nanoparticles

**Figure 3:**
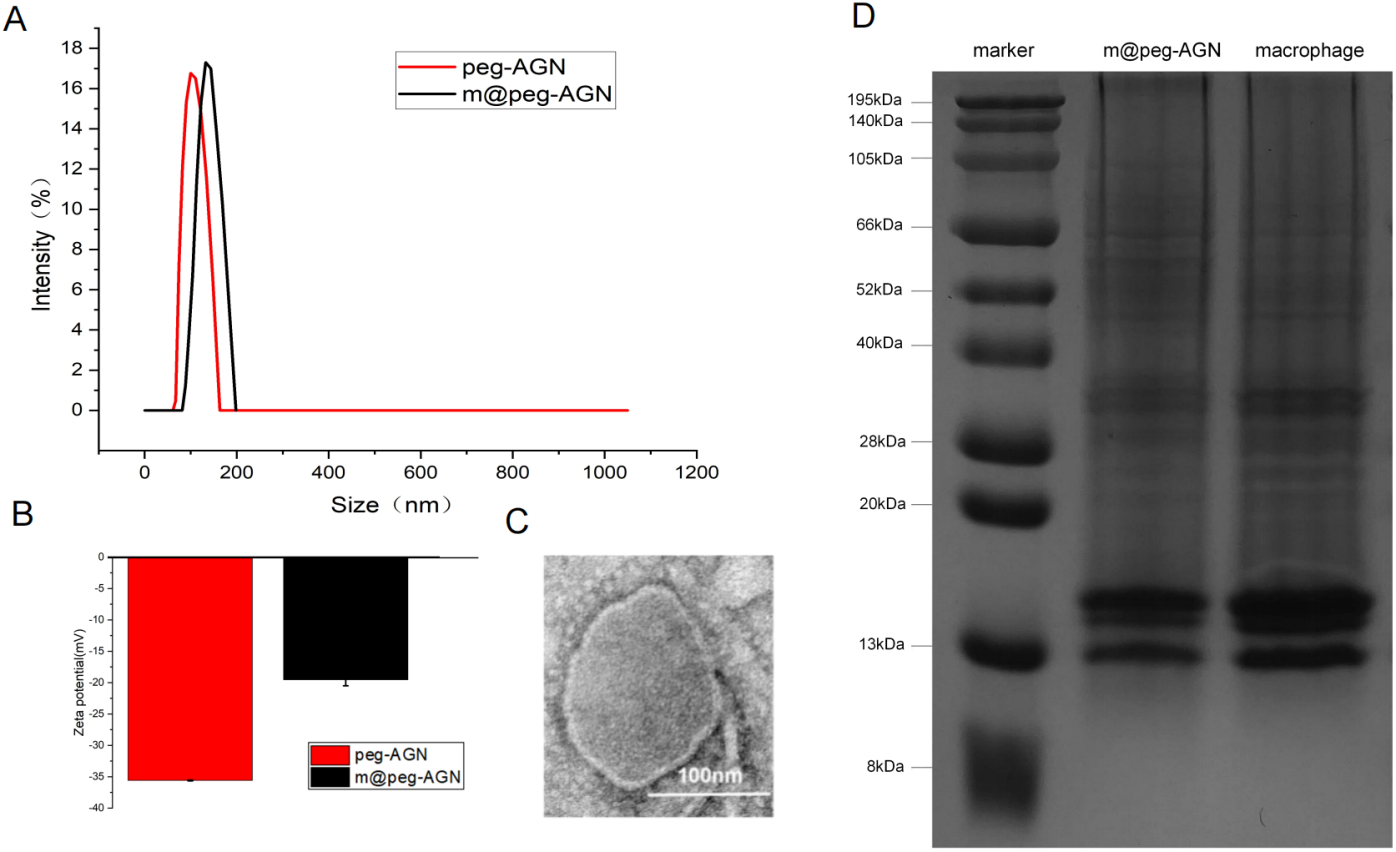
**(A)** Determination of hydration particle size of peg-AGN and m@peg-AGN by dynamic light scattering. **(B)** Zeta potential. **(C)** Transmission electron microscopy (TEM) image of m@peg-AGN. The scale bar in the lower right corner represents 100 nm. **(D)**SDS-PAGE images of macrophage membrane and m@peg-AGN.

Dynamic light scattering (DLS) was utilized to measure the hydrated particle size of peg-AGN, which was approximately 106 nm, and that of m@peg-AGN, which increased to approximately 134 nm after coating (**Figure 3A**). The zeta potential of peg-AGN was about -35 mV, whereas that of m@peg-AGN was approximately -19 mV (**Figure 3B**). The increase in both the hydrated particle size and zeta potential following the coating with macrophage cell membranes (MCM) indicates a successful encapsulation process. Transmission electron microscopy (TEM) further confirmed the presence of a shell structure on the surface of the micelles, visually demonstrating the effective coating of MCM on peg-AGN (**Figure 3C**).

A series of standard AGN solutions at concentrations of 0, 1, 2, 4, 8, and 10 µg/ml were prepared, and their absorbance was measured. Linear regression analysis demonstrated a high degree of linearity in the standard curve, with an R-value of 0.9997(**Supplementary Figure S1D**).

### 3.5 SDS-PAGE analysis and standard curve measurement

The biological functions of macrophage membranes are primarily dependent on the membrane proteins. After treating macrophage membranes and m@peg-AGN samples separately, electrophoresis revealed that the protein bands of m@peg-AGN closely aligned with those of the macrophage membrane, demonstrating that the protein composition of the MCM was well-preserved on the m@peg-AGN surface (**Figure 3D**). This indicates that m@peg-AGN may exhibit similar surface recognition and immune evasion capabilities as macrophages.

### 3.6 Drug loading and release profile of nanocomposites

**Figure 4:**
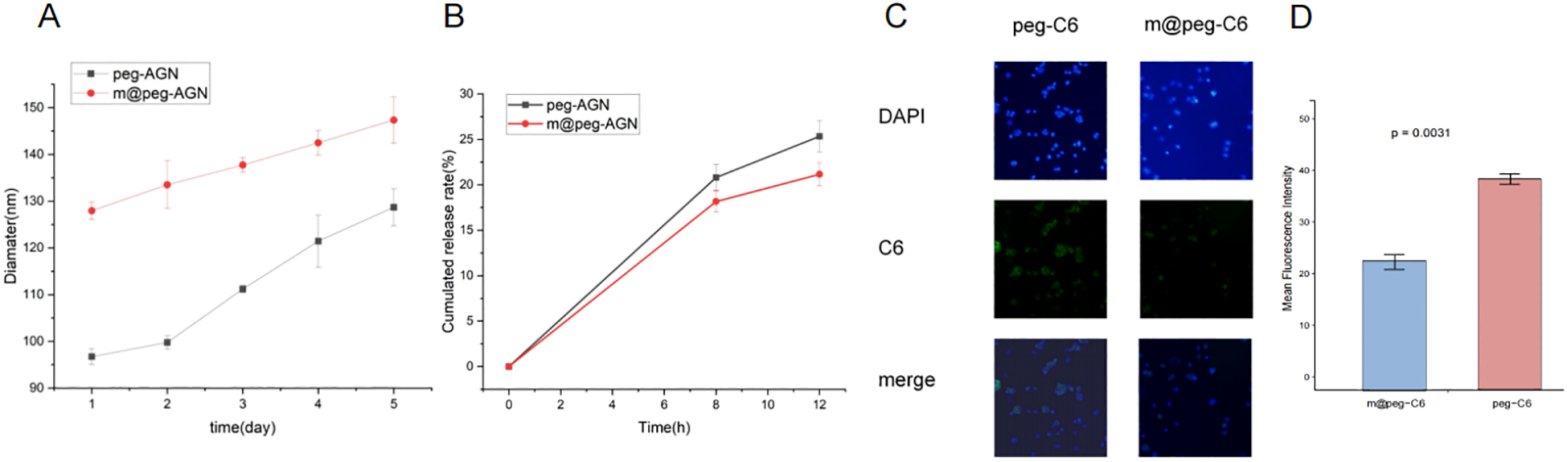
**(A)** The particle size of m@peg-AGN and peg-AGN over a period of five days using DLS. **(B)** Detection of apigenin release performance of m@peg-AGN and peg-AGN. **(C)** The uptake of peg-C6 and m@peg-C6 by macrophages. **(D)** Quantification of mean fluorescence intensity in cells treated with peg-C6 and m@peg-C6 (n=3).

To assess the stability of peg-AGN and m@peg-AGN, both were incubated in PBS solution and their hydrated particle sizes were continuously monitored over a period of five days using DLS (**Figure 4A**). Furthermore, the drug release profiles were evaluated under pH 7.4. After a 12-hour dialysis, approximately 20.7% of AGN was released from m@peg-AGN, compared to about 25% from peg-AGN (**Figure 4B**). This disparity was statistically significant (P < 0.05), indicating that the macrophage membrane coating substantially stabilizes the nanoparticles and moderates the release rate of AGN.

### 3.7 *In vitro* validation of immune evasion capability

Macrophages play a critical role in the clearance of nanoparticles. To investigate the effects of PEG and m@peg nanoparticles (NPs) on macrophage recognition and clearance functions, the unactivated RAW264.7 cell model system was utilized. Fluorescence microscopy facilitated the qualitative analysis of peg-C6 and m@peg-C6 uptake by RAW264.7 cells. After a two-hour treatment, both peg-C6 and m@peg-C6 groups demonstrated weak green fluorescence signals (**Figure 4C**). Notably, signal intensity was lower in the m@peg-C6 group compared to the peg-C6 group. It is indicated that m@peg nanoparticles showed better immune evasion capability. To further quantify these findings, the fluorescence signal intensity was measured, and the data were visualized in **Figure 4D**. The m@peg-C6 group displayed a significantly lower mean fluorescence intensity (p = 0.0031) compared to the peg-C6 group.

### 3.8 Study on the effect of apigenin on the cell cycle of breast cancer cells

**Figure 5:**
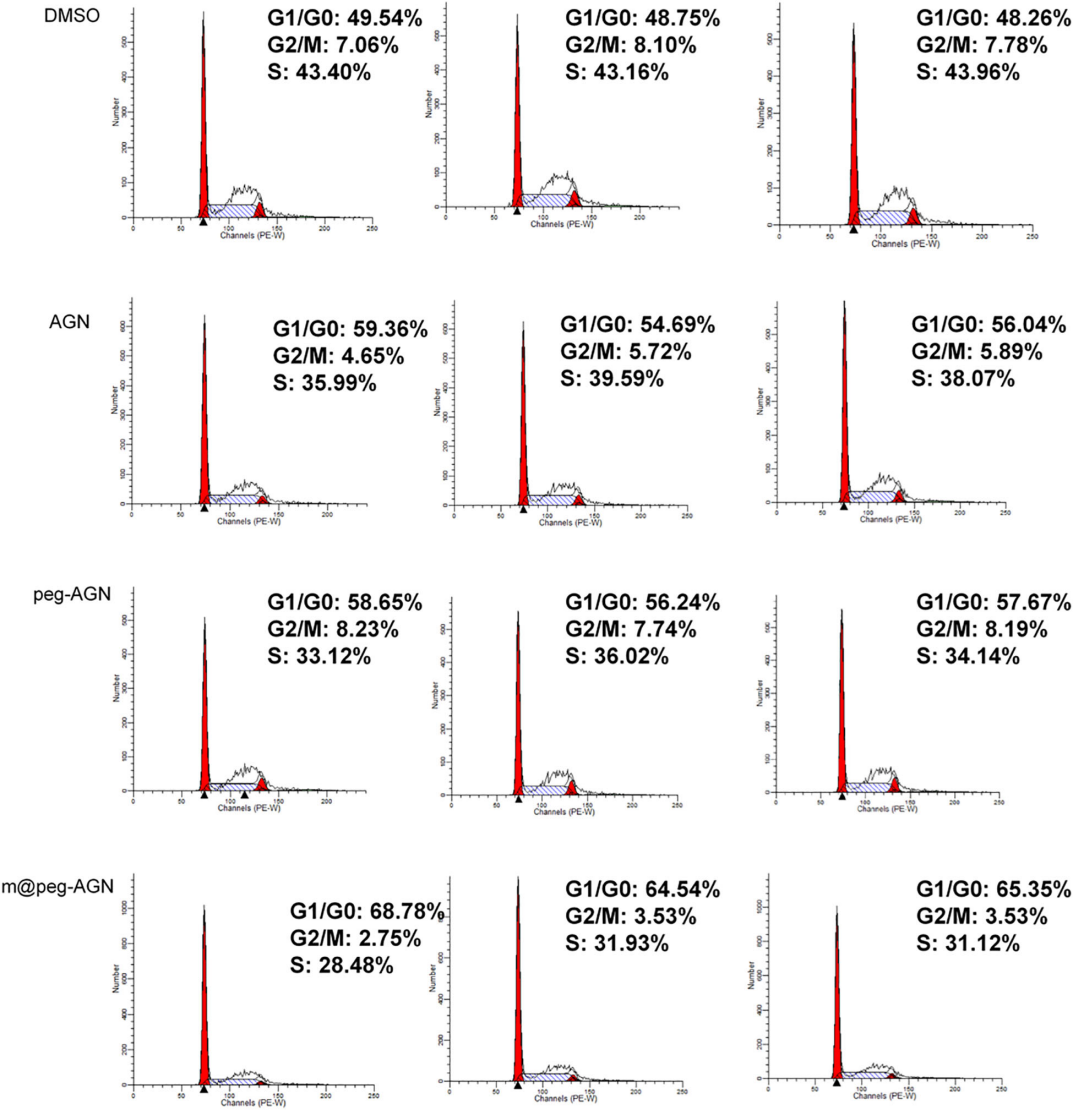
The cell cycle assay of 4T1 cells treated with different AGN formulations.

To further explore the inhibitory effects of apigenin nanoparticles on the proliferation of 4T1 cells, flow cytometry was employed to examine the alterations in various cell cycle phases. Compared to the DMSO control group, cells treated with AGN demonstrated a notable increase in the G0/G1 phase and a significant decrease in the G2/M phase. After treating 4T1 cells with peg-AGN containing 100 µM AGN, it was observed that there was no significant difference of the proportion of cells in the G0/G1 compared to AGN. After treating 4T1 cells with m@peg-AGN,it was observed that the proportion of cells in the G0/G1 phase significantly increased compared to AGN, while the number of cells in the G2/M phase significantly decreased (**Figure 5**). Notably, the effect was more pronounced following treatment with m@peg-AGN. To visualize the distribution of cell cycle phases across different treatment conditions, a stacked bar chart was used (**Supplementary Figure S1C**). The results showed significant differences (p < 0.001) in the distribution of the different treatment groups (DMSO, AGN, peg-AGN, m@peg-AGN) in the G1/G0, G2/M, and S phases of the cell cycle. In particular, the Tukey HSD *post-hoc* test revealed significant differences in the m@peg-AGN-treated group compared with the control group in all phases of the cell cycle, demonstrating that m@peg-AGN was highly statistically significant in altering the cell cycle distribution. In contrast, the effects of AGN and peg-AGN treatments alone were relatively poor (**Supplementary Table S1**, n=3). These data show AGN nanoparticles effectively induce and enhance cell cycle arrest, thereby inhibiting tumor cell proliferation.

### 3.9 *In vivo* biodistribution and targeting evaluation of m@peg NPs

**Figure 6:**
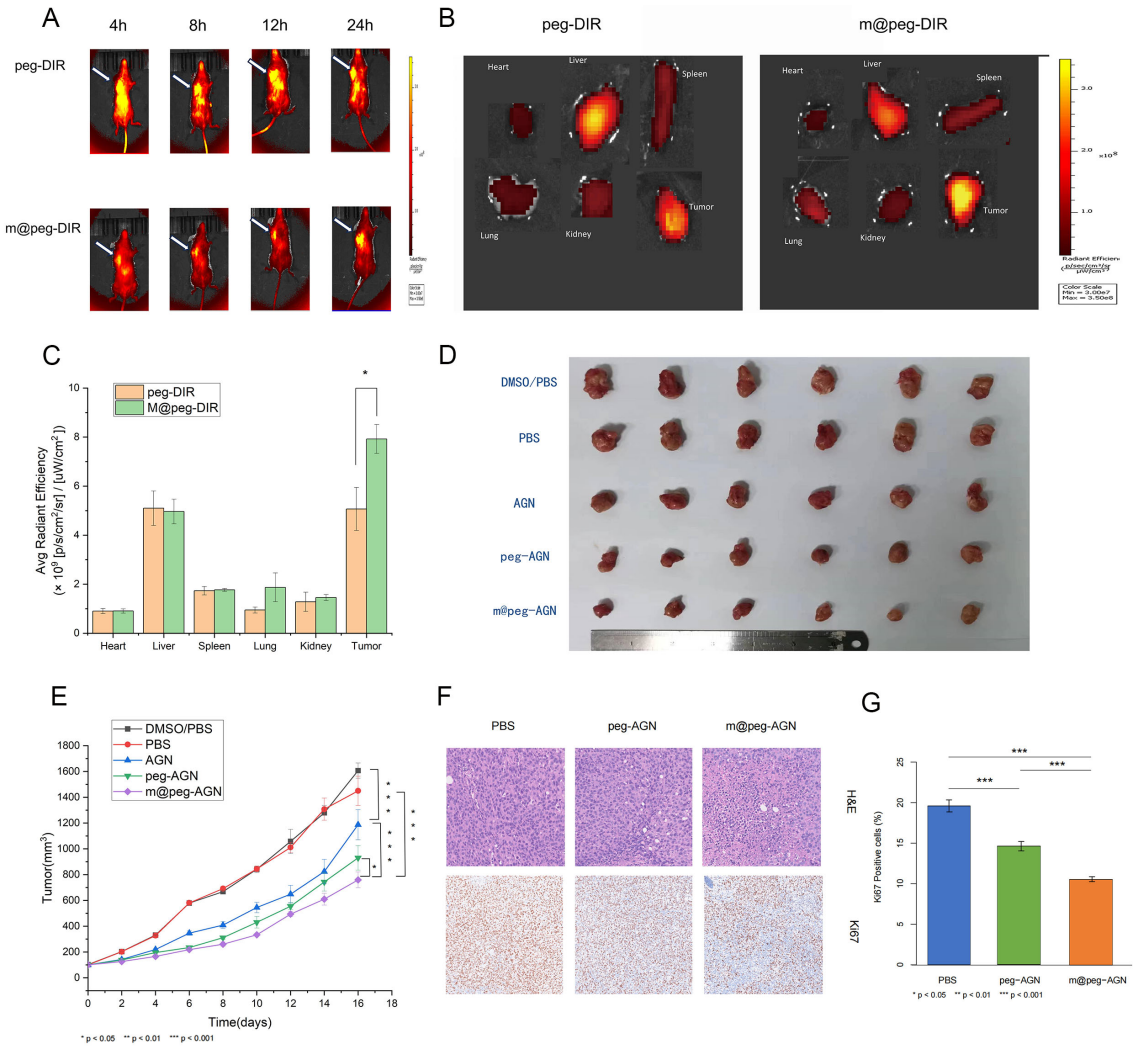
**(A)** Fluorescence imaging of tumor bearing mice. **(B)** *In vitro* tissue fluorescence imaging. **(C)** Quantitative analysis of fluorescence uptake by different tissues. **(D)** White light photos of tumor resection after different treatments. **(E)** Tumor growth curves of different treatment methods. **(F)** H&E staining and Ki67 staining of tumors in each group of mice. Scale=100um. **(G)** Quantification of Ki-67 positive cells (%) in tumor tissues from different groups (n = 3).

Small animal fluorescence revealed more DIR signal at the tumor site in the m@peg-DIR group within 24 hours. While the peg-DIR group displayed a similar pattern, the accumulation was less significant in comparison to the m@peg-DIR group with no significant difference of accumulation in the liver between the two groups. These results suggest that the macrophage membrane camouflage in m@peg-DIR enhances its drug delivery (**Figures 6A–C**).

### 3.10 *In vivo* antitumor efficacy of m@peg-AGN nanoparticles

Following 16 days of treatment, there was a noticeable reduction in tumor volume in the mice treated with free AGN compared to the control group. Significantly, the m@peg-AGN group exhibited the most pronounced antitumor effect, evidencing better tumor growth inhibition relative to the other groups(**Figures 6D, E**). That demonstrates m@peg-AGN could be a potential therapeutic drug for TNBC.

Further analysis through H&E staining revealed an increase in necrotic cells in the tumor tissues of mice treated with m@peg-AGN compared to the control groups. Additionally, Ki-67 staining indicated a lower tumor proliferation rate in the m@peg-AGN group (**Figure 6F**). In contrast, the percentage of area with positive Ki-67 in the m@peg-AGN group (10.56 ± 0.28%) was significantly decreased compared to the PBS (19.60 ± 0.60%) and peg-AGN (14.61 ± 0.44%) groups, as shown in (**Figure 6G**) (p < 0.001).

### 3.11 *In vivo* biosafety evaluation

**Figure 7:**
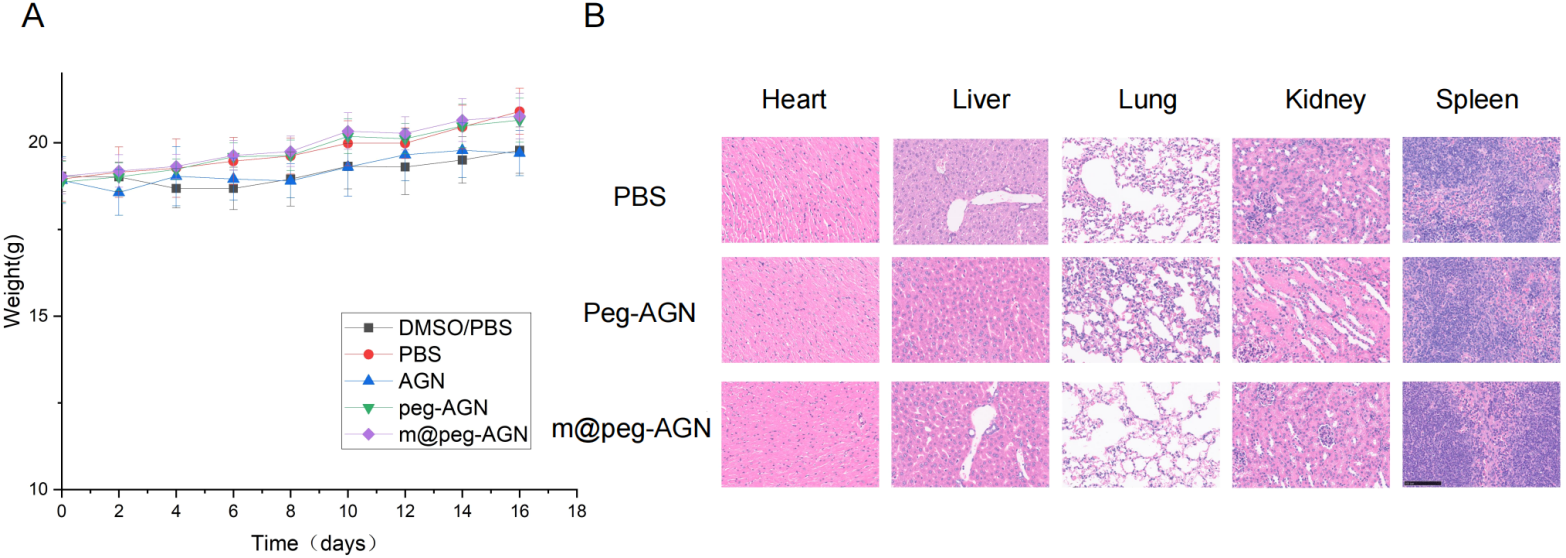
**(A)** Weight monitoring of mice in each group. No statistically significant differences in body weight among the groups throughout the experimental period. **(B)** H&E stained sections of the main organs of tumor bearing mice.

After 16 days of meticulous observation and detailed recording, no significant differences in living conditions or body weight were observed among the five groups of mice (**Figure 7A**). After sixteen days, the major organs of these mice were harvested and subjected to histological examination using H&E staining. The findings revealed that, in comparison to the PBS group, the AGN nanoparticles did not induce significant pathological damage to major organs, including the heart, liver, spleen, lungs, and kidneys (**Figure 7B**). Notably, the free AGN group was excluded from this study because of its poor water solubility and the potential organ damage that could result from DMSO.

## 4 Discussion

In this study, network pharmacology was utilized to elucidate the tumor inhibitory mechanisms and molecular actions of apigenin in TNBC treatment. Network pharmacology has emerged as a powerful tool for understanding the complex interactions between diseases and drugs, thus aiding in development of therapeutic drugs (19, 20). It provides substantial scientific support for developing and applying traditional Chinese medicine (21). In our study, 21 key genes were identified, providing insights into the molecular mechanisms of AGN in TNBC. Protein-protein interaction network and pathway enrichment analyses revealed critical genes (CDH1, TP53, CTNNB1, CCND1, CASP8) involved in TNBC onset and progression, as well as pathways essential for AGN’s therapeutic action. These findings underscore the potential of AGN as a treatment drug for breast cancer.

Among the significantly enriched Gene Ontology (GO) terms, protein kinase activity, transcription factor binding, chromatin binding, and cyclin-dependent protein serine/threonine kinase activity possibly play an important role in pathogenesis and progression of breast cancer. One group revealed the involvement of Bax, PARP proteins in AGN mediated apoptosis in MDA MB-231 cells (22). Other group has concluded AGN induces caspase-dependent apoptosis by inhibiting signal transducer and activator of transcription 3 signaling in HER2-overexpressiong SKBR3 breast cancer cells (23). All these studies could support the possible mechanisms from GO terms plays part in the regulation of cell growth and apoptosis.

A novel biomimetic nanodrug platform, incorporating macrophage membranes (m@peg-AGN), was designed to enhance immune evasion and improve the delivery of AGN. *In vitro* experiments demonstrated that the macrophage membrane coating effectively reduced immune recognition and clearance, significantly improving drug efficacy and tumor growth inhibition. As discussed by Chen et al. (24), liposomal systems have demonstrated good targeting efficiency, but our findings suggest that the macrophage membrane coating in m@peg-AGN nanoparticles has improved biodistribution in the m@peg-DIR group. Gong et al. (25) demonstrated that macrophage membrane coatings help nanoparticles evade immune surveillance. Since our study shows enhancing anti-tumor efficacy, but our results didn’t support a similar reduction in immune clearance. Although our study did not directly assess the interaction between m@peg-AGN nanoparticles and tumor-associated macrophages (TAMs), it is possible that the macrophage membrane coating facilitates interactions with TAMs in the tumor microenvironment, which could further enhance immune evasion and contribute to the nanoparticles’ efficacy in inhibiting tumor growth. Future studies should investigate the direct interaction between these nanoparticles and TAMs using techniques such as flow cytometry or immunofluorescence to explore whether these interactions play a role in modulating the immune response in the tumor microenvironment.

In the vitro study of validation of immune evasion capability, suggesting that the macrophage membrane coating partially inhibits the recognition and clearance functions of the mononuclear phagocyte system. This enhanced immune evasion improved the nanoparticles’ ability to penetrate tumor cells and inhibit their migration. The results demonstrated that peg-AGN, compared to free AGN, penetrated tumor cells more efficiently and exhibited stronger inhibition of 4T1 cell migration. Furthermore, owing to the tumor-targeting properties of macrophages, m@peg-AGN significantly enhanced cellular uptake and further reduced the migratory capacity of 4T1 cells.

Recent studies have highlighted the antitumor properties of AGN, particularly the ability to modulate the cell cycle in cancer cells. In the previous finding, treatment of AGN in MCF-7 cells followed by induction of G2/M phase cell cycle (26). Another group found an increase in the sub G_0_/G_1_ apoptotic population in SKBR3 cells after treated with AGN (23). The difference we observed is growth -suppressive activity of AGN accompanied by a significant increase in the G0/G1 phase and a notable reduction in the S phase at a concentration of 100 µM AGN, in comparison to the DMSO control group. The difference about the distribution of cell cycle may be due to different breast cancer cell lines. Furthermore, when AGN is delivered via nanoparticles, its pharmacokinetics and cellular uptake are greatly enhanced. This suggests that AGN may effectively induce cell cycle arrest, thereby inhibiting tumor cell proliferation. Compared with the free AGN and peg-AGN, the proportion of G1/G0 phase cells in m@peg-AGN increased significantly, while the number of G2/M and S phase cells decreased significantly. As discussed in the research, the nanoparticles not only improve drug solubility and stability but also facilitate lysosomal escape (27). The ability of these nanoparticles to escape lysosomes means that AGN can be released directly into the cytoplasm, where it can exert a more potent effect on cellular processes, including those regulating the cell cycle. Therefore, the modulation of the cell cycle by AGN nanoparticles not only provides critical insights into their mechanism of action but also supports their potential as a therapeutic strategy for breast cancer treatment.

In the 4T1 tumor-bearing mouse model, m@PEG-AGN exhibited the better tumor growth inhibition, comparing with PEG-AGN and free AGN. Furthermore, H&E staining and Ki-67 immunohistochemical analysis confirmed the strong antitumor effect of m@PEG-AGN. These findings provide strong experimental evidence supporting the use of apigenin nanoparticles for antitumor applications *in vivo*. Mechanistically, the macrophage membrane coating on m@peg nanoparticles improved the solubility and stability of AGN, effectively preventing immune clearance and ensuring sustained therapeutic action within tumor tissues. Safety evaluations indicated that m@peg-AGN caused no damage to major organs, demonstrating excellent biocompatibility. Based on the above findings, it can be concluded that m@peg-AGN is a nanotherapeutic agent with excellent biosafety, making it a promising alternative to conventional free drugs and offering new possibilities for clinical treatment.

However, for clinical translation, several important factors must be addressed. First, the scalability of the manufacturing process for m@peg-AGN nanoparticles needs to be optimized to meet the demand for large-scale production. This includes ensuring the consistency, quality control, and reproducibility of the nanoparticle formulation. Second, although the safety profile of m@peg-AGN is promising, further studies on its long-term toxicity and potential side effects are necessary, particularly regarding chronic exposure and the potential for immune system interaction. Finally, regulatory concerns related to nanoparticle-based therapeutics must be carefully considered. This includes meeting regulatory standards for drug delivery systems and ensuring that the nanoparticle formulation is safe, effective, and compliant with existing regulatory frameworks.

Our research has certain limitations. Only one TNBC cell line (4T1) was tested in this study, and further validation in other TNBC cell lines, such as MDA-MB-231 and BT-549, would be valuable to improve the robustness and broader relevance of the results. Moreover, although macrophage membrane-coated nanoparticles show promise in preclinical models, their use in humans may raise immunological concerns, such as immune activation or tolerance. In addition, the absence of long-term toxicity studies, including investigations into immune responses and nanoparticle clearance, is a significant limitation. These factors are crucial for fully understanding the safety profile of the nanoparticles. Comprehensive immunological evaluations will be required to assess these potential issues. Finally, our study only evaluates single-modality therapy using m@peg-AGN nanoparticles. Future studies should explore the combination of AGN with other therapies, such as chemotherapy or checkpoint inhibitors, to investigate potential synergistic effects and enhanced therapeutic efficacy. For instance, previous studies have shown that combining natural compounds like luteolin and apigenin with immune checkpoint inhibitors can improve anti-tumor immunity and boost therapeutic outcomes (28). This approach could provide valuable insights into the clinical potential of AGN nanoparticles and expand their application in combination with other cancer treatments.

## 5 Conclusion

In summary, this study employed network pharmacology to identify potential therapeutic targets and mechanisms of AGN in breast cancer treatment. A novel AGN nano-delivery system (m@peg-AGN) was developed, utilizing biomimetic nanotechnology to enhance AGN’s solubility, reduce immune clearance, and improve drug delivery to the tumor site. The system demonstrated significant anti-tumor effects *in vivo*, moderating cell cycle and advancing the treatment of TNBC. The practical implications of this research suggest that m@peg-AGN could serve as a targeted therapy for TNBC, providing an alternative to conventional treatments. Future research should focus on further *in vivo* studies to evaluate the long-term safety and efficacy of m@peg-AGN and investigate its potential in combination with other therapeutic agents. Additionally, exploring the mechanisms of immune evasion and tumor targeting will be crucial to maximizing the therapeutic benefits of this delivery system.

## Data Availability

The raw data supporting the conclusions of this article will be made available by the authors, without undue reservation.

## Supplementary material

Supplementary Figure 1
**(A)** Gene Ontology Enrichment Analysis of Intersection Genes. **(B)** KEGG pathway analysis of intersecting genes. **(C)** Cell cycle distribution of cells treated with DMSO, AGN, peg-AGN, and m@peg-AGN. **(D)** Determination of AGN standard curve.

Supplementary Table 1 Effects of apigenin and its nanoparticles on cell cycle distribution of 4T1 breast cancer cells.
