# Supplementary figures and images for: Development of a biomimetic nanoparticle platform for apigenin therapy in triple-negative breast cancer
Source: Front Oncol. 2025 May 16;15:1521529. doi: 10.3389/fonc.2025.1521529 (PMC12122512; doi:10.3389/fonc.2025.1521529)

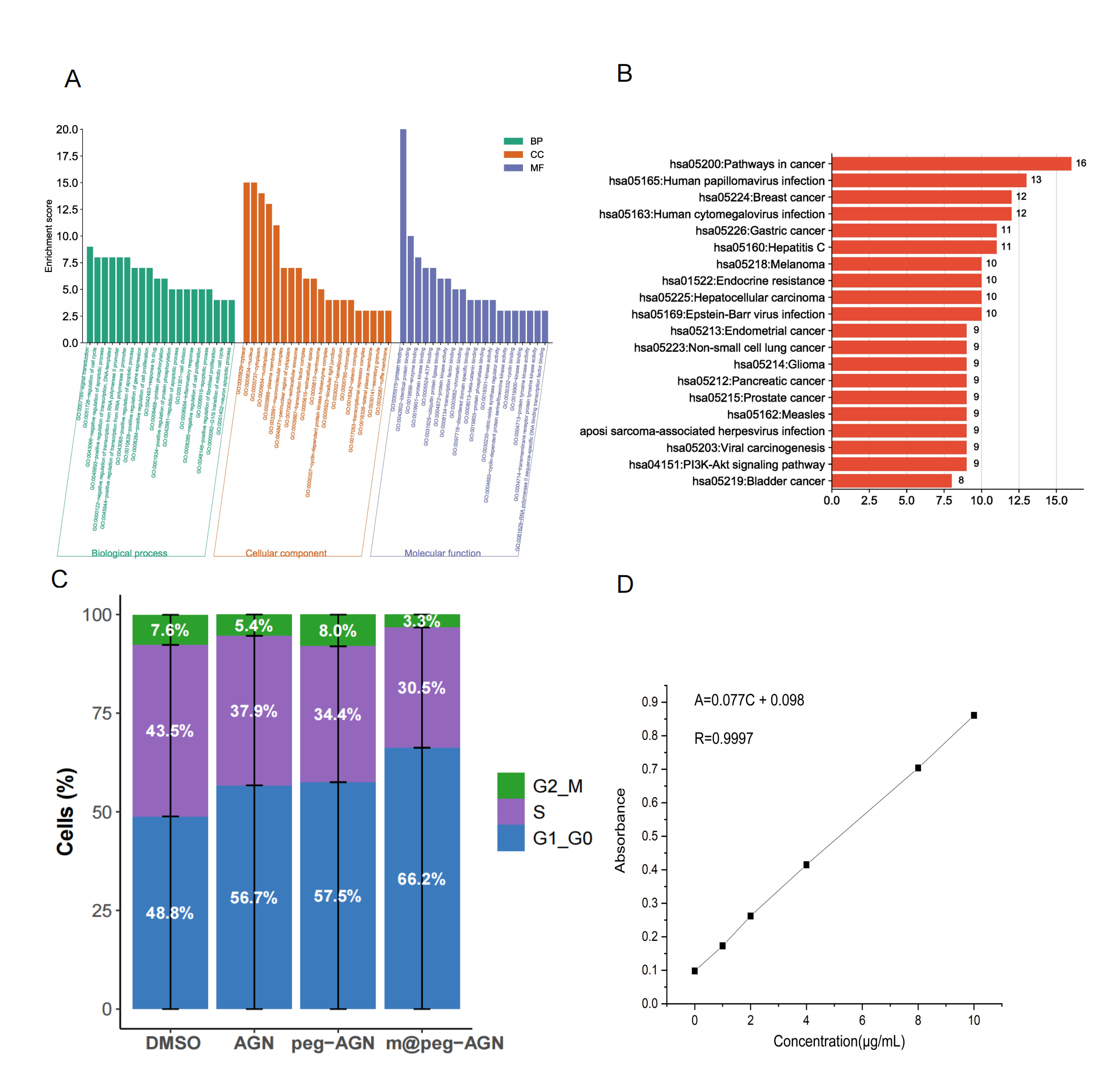

Supplement: Supplementary Figure 1 — (A) Gene Ontology Enrichment Analysis of Intersection Genes. (B) KEGG pathway analysis of intersecting genes. (C) Cell cycle distribution of cells treated with DMSO, AGN, peg-AGN, and m@peg-AGN. (D) Determination of AGN standard curve. [file Image1.tif]
